# The efficiency of routine infant immunization services in six countries: a comparison of methods

**DOI:** 10.1186/s13561-019-0259-1

**Published:** 2020-01-08

**Authors:** Nicolas A. Menzies, Christian Suharlim, Stephen C. Resch, Logan Brenzel

**Affiliations:** 1 Department of Global Health and Population, Harvard T.H. Chan School of Public Health, 665 Huntington Ave, Boston, MA 02115 USA; 2 Center for Health Decision Science, Harvard T.H. Chan School of Public Health, Boston, USA; 3 Bill & Melinda Gates Foundation, Seattle, Washington USA

**Keywords:** Efficiency, organizational, Costs and cost analyses, Immunization programs, Vaccination

## Abstract

**Background:**

Few studies have systematically examined the efficiency of routine infant immunization services. Using a representative sample of infant immunization sites in Benin, Ghana, Honduras, Moldova, Uganda and Zambia (316 total), we estimated average efficiency levels and variation in efficiency within each country, and investigated the properties of published efficiency estimation techniques.

**Methods:**

Using a dataset describing 316 immunization sites we estimated site-level efficiency using Data Envelopment Analysis (DEA), Stochastic Frontier Analysis (SFA), and a published ensemble method combining these two approaches. For these three methods we operationalized efficiency using the Sheppard input efficiency measure, which is bounded in (0, 1), with higher values indicating greater efficiency. We also compared these methods to a simple regression approach, which used residuals from a conventional production function as a simplified efficiency index. Inputs were site-level service delivery costs (excluding vaccines) and outputs were total clients receiving DTP3. We analyzed each country separately, and conducted sensitivity analysis for different input/output combinations.

**Results:**

Using DEA, average input efficiency ranged from 0.40 in Ghana and Moldova to 0.58 in Benin. Using SFA, average input efficiency ranged from 0.43 in Ghana to 0.69 in Moldova. Within each country scores varied widely, with standard deviation of 0.18–0.23 for DEA and 0.10–0.20 for SFA. Input efficiency estimates generated using SFA were systematically higher than for DEA, and the rank correlation between scores ranged between 0.56–0.79. Average input efficiency from the ensemble estimator ranged between 0.41–0.61 across countries, and was highly correlated with the simplified efficiency index (rank correlation 0.81–0.92) as well as the DEA and SFA estimates.

**Conclusions:**

Results imply costs could be 30–60% lower for fully efficient sites. Such efficiency gains are unlikely to be achievable in practice – some of the apparent inefficiency may reflect measurement errors, or unmodifiable differences in the operating environment. However, adapted to work with routine reporting data and simplified methods, efficiency analysis could triage low performing sites for greater management attention, or identify more efficient sites as models for other facilities.

## Background

Given constrained budgets, increasing the efficiency of health service delivery is seen as a way to free up resources for increasing the scale and quality of services [1]. Improving efficiency is not among the goals described by the WHO’s Global Vaccine Action Plan [2], yet doing so could make funds available to address immunization program objectives, such as increasing population coverage and introducing new vaccines. A variety of approaches have been used to investigate the level of technical efficiency achieved by immunization programs. One general class of approaches can be considered ‘program review’, involving observation and interviews with program personnel, consultation of stakeholders, and detailed record review. These approaches are able to draw on a wide range of evidence, and produce qualitative findings that may cover a much wider scope than just technical efficiency [3–6]. Intensive approaches such as the WHO’s National Immunization Programme Reviews [7] may reveal areas where resources are being wasted, or where underinvestment in some program components is hampering overall performance. These reviews are intended to not only diagnose problems but also suggest solutions, and consider both implementation and strategic challenges. However, these approaches can have limited reproducibility, given the central role of the investigator in evidence discovery and interpretation.

Other approaches for investigating efficiency rely on the quantitative comparison of inputs (e.g. resources consumed) and outputs (e.g. services provided), for a sample of service providers. One approach focuses on mean values, either estimating the overall average cost per output, or using stratification or regression methods to estimate this average for subsets of the sample. With this approach variation in efficiency within the program is described with respect to the mean, and conclusions about program-level efficiency drawn by comparing the mean estimate to external standards [8–12]. A third class of approaches attempts to estimate efficiency directly. These ‘frontier approaches’ estimate an efficient frontier describing sites operating at peak efficiency, and then compare all other sites to this standard [13, 14]. Program-level efficiency can be gauged by constructing a frontier across comparable programs [15]. All these quantitative approaches make the assumption that other service providers—particularly those that are similar in terms of relevant characteristics—can be used to create a benchmark for assessing efficiency.

Use of frontier approaches for efficiency evaluation within healthcare is growing [16–18], yet there are few examples of applications for immunization programs [19–21]. In one of few examples, Hollingsworth et al. compared 23 facilities providing immunization services in Victoria, Australia, finding modest evidence that urban sites were operating more efficiently than their rural peers. In a larger study, Valdmanis and colleagues examined the efficiency of 117 vaccination sites operating in Dhaka, Bangladesh, finding that sites were generally operating at a low level of efficiency, with greater efficiency statistically associated with government ownership, fixed location (as compared to outreach clinics), and greater time since site operations began. This study also found clear evidence of scale economies associated with higher service volume [20].

In this analysis we draw on a unique data set of immunization service costs collected as part of the EPIC studies, funded by the Bill & Melinda Gates Foundation to fill the knowledge gap around immunization costs and financing. These data describe the costs and performance of a large, representative sample of immunization sites in Benin, Ghana, Honduras, Moldova Uganda and Zambia [22–24]. Using these data, we investigate different quantitative approaches for estimating the efficiency of immunization sites, to describe their relative performance and draw conclusions about their utility for efficiency evaluation within infant immunization programs, as well as provide summary estimates of efficiency at a country level and describe within-country variation in efficiency.

## Methods

### Data and sample

The EPIC studies collected information on service volume, costs and other characteristics for 319 sites providing routine immunization services in Benin, Ghana, Honduras, Moldova, Uganda, and Zambia during the 12-month period January–December 2011 [22]. Data were collected through a series of country-level studies implemented during 2012–13 [25–30]. Sites were selected from a sampling frame consisting of public and NGO facilities providing routine immunization services, and data were collected using a standardized approach [31], to allow comparison and pooling of data across sites and settings.

For this analysis, we examined variation in the efficiency of service provision between participating sites. Data from all six country studies were cleaned by a central project team, and variable definitions standardized. We removed sites with anomalous or missing values for key variables (3 sites), for an analytic sample of 316 sites. All data are freely available for download at www.immunizationcosting.org, along with study materials and publications [32].

### Efficiency measure

We operationalize efficiency as the Shephard input efficiency [33]. This measure describes the value by which the inputs of a particular site would need to be multiplied in order to move that site onto the efficient frontier, holding outputs constant. Under this definition, the efficiency score is bounded between zero and one, with higher values indicating greater efficiency. Input efficiency was used in preference to output efficiency (which considers hypothetical changes in output, holding inputs constant), as substantial increases in service delivery volume may be implausible for sites that are serving close to 100% of their potential target population.

### Inputs and outputs

In the main analysis, inputs were represented by the total service delivery costs of immunization services provided by the facility. This includes all resources expended for site-level immunization activities (staff salaries, per diems, volunteer incentives, transport, buildings, utilities, equipment, and non-vaccine consumables). We excluded vaccine and vaccine supplies, as these may be less sensitive to site-level decisions, and show low variation across sites per unit of output [23]. Administrative costs incurred by district, regional, and central offices were also excluded from the analyses. Outputs were represented by the total children receiving DTP3 (3rd dose of diphtheria, tetanus, and pertussis, typically via a single injection of the pentavalent vaccine), a conventional proxy for completion of the basic infant immunization schedule.

### Analytic approach

We used three methods for estimating the efficient frontier (DEA, SFA, ensemble method). For each of these methods we calculated the Shephard input efficiency of individual sites in comparison to the estimated frontier. As a forth comparator we constructed a simplified efficiency index using the residuals derived from a conventional production function fitted to site input and output data. Details for each of these methods are provided below.

#### Data envelopment analysis

Data Envelopment Analysis (DEA) uses a non-parametric linear programming approach [14] to estimate the efficient frontier for a sample of discrete decision-making units, which in this study are represented by immunization service delivery sites. The frontier is constructed as a piecewise linear function joining the most efficient sites, and describes the minimum set of inputs required to produce a given set of outputs. DEA allows relatively weak assumptions to be made about the shape of the efficient frontier, and for this analysis we allowed for variable returns-to-scale, consistent with earlier studies [20]. DEA estimates are sensitive to outliers, which could reflect measurement error or features of the sites operating environment making them incomparable to other sites. We used super-efficiency analysis to identify and remove outliers, calculating efficiency for each site based on a frontier constructed from the other sites [34]. This produces scores > 1.0 for the most efficient sites, and we removed the site with the highest score above 1.5. We repeated this procedure until no score exceeded 1.5 or 5% of sites had been removed [35]. Super-efficient sites were assigned a Shephard input efficiency of 1.0 and not used for estimating the efficient frontier. For the other sites, the Shephard input efficiency was calculated with respect to the efficient frontier estimated from the non-excluded sites. Using conventional DEA methods can overestimate the efficiency of sites, particularly where the sample is small. We corrected for this using the Simar and Wilson parametric bootstrapping approach [36] implemented with 5000 bootstrap replicates.

#### Stochastic frontier analysis

Under Stochastic Frontier Analysis (SFA), the efficient frontier is estimated by fitting a parametric production function (e.g. Cobb-Douglas), in a model that assumes deviations from the regression line can be decomposed into the sum of a mean-zero error term representing measurement error in the output plus a one-sided term representing deviations from efficient production. This formulation allows the efficient frontier to be constructed as a translation of the fitted regression line [13]. As the approach allows for measurement error, not all sites need to fall within this frontier. The use of a parametric function for mean output is seen as a drawback of this approach and can artificially constrain the form of the frontier. To relax this constraint we used the semi-parametric approach developed by Fan et al. [37], using thin plate regression splines (*f*() in eq. 1) in place of a parametric mean function [38]. Equation 1 shows the relationship that was estimated, where *Y*_*i*_ and *X*_*i*_ represent total outputs and inputs for site *i,* respectively, *f*() represents the flexible spline used to model the relationship between inputs and outputs, *υ*_*i*_ represents the error term, and *μ*_*i*_ represents the efficiency term. Following common practice, we assumed a Normal distribution for the error term *υ*_*i*_ and a half-Normal distribution for the efficiency term *μ*_*i*_, and estimated the regression using logged inputs and outputs. As a consequence of these assumptions the regression residuals (*υ* − *μ*) are assumed to be left-skewed, and valid efficiency estimates cannot be calculated if this assumption does not hold. We calculated the Shephard input efficiency from the estimated values for *μ*_*i*_.

1 $$ \mathit{\ln}\left({Y}_i\right)=f\left(\mathit{\ln}\left({X}_i\right)\right)+{\upsilon}_i-{\mu}_i $$

#### Ensemble methods

Several studies have investigated the relative benefits of SFA and DEA estimators for different applications [39]. Directly relevant to this analysis is a recent study by Di Giorgio et al. [35] who investigated SFA and DEA estimators using simulated data on health services delivery costs in low and middle income settings. This study found that efficiency estimates from both methods were unsatisfactory in the presence of non-trivial measurement error, particularly when distributional assumptions were not met. The relative performance of the two approaches was dependent on the shape of the production function, but the authors found an ensemble approach, averaging both estimators, was preferred across a wide range of scenarios. We applied this ensemble approach as a third efficiency estimator.

#### Ordinary least squares

For each country we fitted a conventional production function to input and output data via ordinary least squares (OLS) [40]. Equation 2 shows the relationship that was estimated, where *Y*_*i*_ and *X*_*i*_ represent total outputs and inputs for site *i,* respectively. The relationship between inputs and outputs was modelled using intercept, linear and quadratic terms, and the regression equation estimated using logged inputs and outputs.

2 $$ \mathit{\ln}\left({Y}_i\right)={\beta}_0+{\beta}_1\mathit{\ln}\left({X}_i\right)+{\beta}_2\mathit{\ln}{\left({X}_i\right)}^2+{\varepsilon}_i $$

We used the residuals from this regression as a simplified efficiency index, to provide a less technically-demanding comparator for the frontier methods described above. This is similar to the Corrected OLS (COLS) approach [41], where the fitted regression line from a conventional production function is shifted upwards until it passes through the most extreme observation, and this shifted line used to represent the efficient frontier. In our implementation we do not estimate this COLS frontier, in order to retain a method based solely on conventional regression techniques.

With all these methods it is assumed the sample of sites being compared is sufficiently homogenous, i.e. undertaking comparable activities, with comparable resources, to produce comparable products, while operating in comparable environments [42]. While many aspects of immunization service provision are similar across countries, it is likely the operating environment differs substantially, and it is unlikely the performance of immunization sites in one country can be used to form an efficient frontier for sites in another. For this reason, we estimated efficiency scores on a country-by-country basis.

### Sensitivity analysis

We assessed the consistency of the results for different formulations of inputs (using total site-level costs including vaccines, site-level vaccine wastage, and vaccine supplies, and excluding above-site supply-chain costs) and outputs (using total infant doses delivered in place of DTP3). For each approach we calculate the lambda statistic proposed by Badunenko et al. as an indicator of the reliability of the efficiency estimator [43], and used Spearman’s rank correlation coefficient to describe agreement between the site efficiency rankings created by each estimator.

### Implementation

Analyses were conducted in R [44]. The Simar and Wilson DEA estimator was implemented using the FEAR package [45]. The Fan et al. SFA estimator was implemented using the semsfa package [46]. Results reported for sample means, variances and distributions were adjusted for survey weighting.

## Results

### Distribution of inputs and outputs

Table 1 presents summary statistics for inputs, outputs, and the cost-per-output for each country. There was substantial variation within and between countries for each outcome, and all outcomes were positively skewed.

**Table 1:** Average facility-level inputs, outputs, and costs per output^a^

Outcome	Benin	Ghana	Honduras	Moldova	Uganda	Zambia
Number of facilities in sample	45	50	71	50	49	51
Service delivery cost^b^ (000 s)	5.55 (2.21)	12.4 (7.83)	7.66 (8.85)	3.72 (7.47)	4.70 (5.75)	19.0 (11.1)
Total cost^c^ (000 s)	18.0 (9.53)	17.9 (11.7)	13.4 (19.0)	4.27 (8.82)	8.00 (11.0)	27.9 (20.7)
DTP3^b^	601 (417)	321 (306)	105 (200)	30.8 (73.9)	298 (667)	702 (904)
Doses	6540 (4210)	2930 (2940)	1420 (3140)	313 (773)	2890 (5790)	6880 (10100)
Service delivery cost per DTP3	12.3 (6.7)	82.6 (130)	165 (154)	191 (131)	28.8 (21.1)	52.7 (40.7)
Total cost per DTP3	35.2 (15.7)	106 (137)	223 (169)	210 (135)	40.1 (20.9)	64.9 (40.9)
Service delivery cost per dose	1.11 (0.705)	9.77 (15.2)	12.2 (10.1)	16.7 (8.16)	3.52 (3.98)	5.75 (4.2)
Total cost per dose	3.16 (1.33)	12 (15.7)	16.6 (10.3)	18.5 (8.24)	4.69 (4.08)	7.07 (4.23)

^a^Results estimated from analysis of the cleaned pooled dataset of 316 sites. Values in parentheses represent sample standard deviation. All estimates adjusted for survey weighting. All costs reported in 2011 USD

^b^Outcomes used in main analysis

^c^Total costs include site-level service delivery costs (staff salaries, per diems, volunteer incentives, transport, buildings, utilities, equipment, and non-vaccine consumables) as well as vaccines and vaccine supplies [23]

### Efficient frontiers estimated by DEA and SFA

**Fig. 1:**
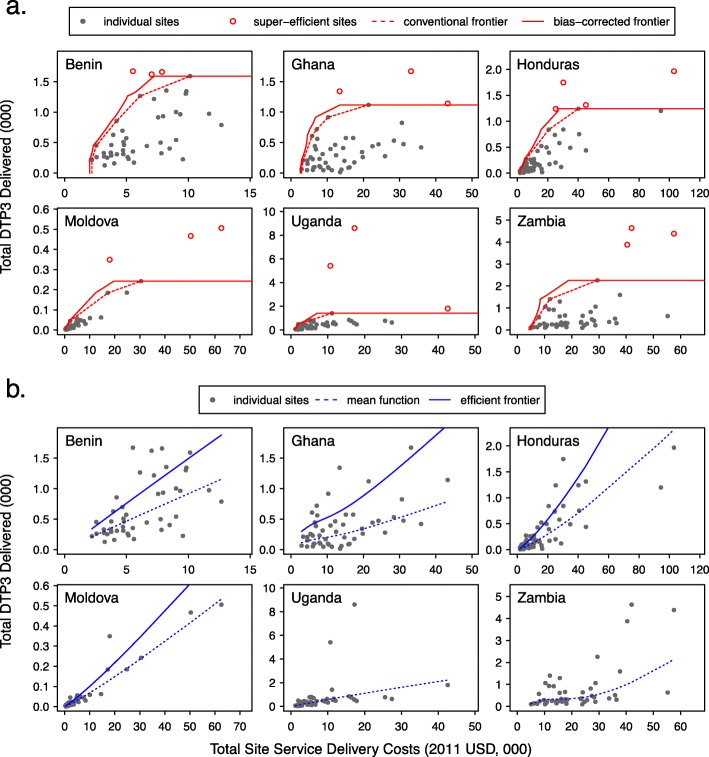
Efficient frontiers estimated by DEA and SFA. Panel **a** efficient frontier estimated by DEA under variable returns-to-scale for each country. Dashed line represents the conventional deterministic DEA frontier. Solid line represents the Simar and Wilson bias-corrected DEA frontier. Panel **b** efficient frontier estimated by SFA for each country. Dashed and solid lines represent the mean function and efficient frontier respectively, for the semi-parametric SFA estimator proposed by Fan et al. For Uganda and Zambia no frontier could be calculated, due to inability to decompose error and efficiency terms

Panel A of Fig. 1 presents plots of the efficient frontier estimated by DEA for each country. Frontiers produced by conventional and Simar and Wilson bias-corrected estimators are shown, with the latter consistently lying to the right of the conventional estimator. Sites excluded by super-efficiency analysis are highlighted in red, and in general these sites lie far from the center of the distribution in each county, with substantially greater service volume. For each country the efficient frontier is concave, reflecting the assumption that if two input-output combinations are feasible, then any linear combination of those input-output combinations is also feasible. Panel B of Fig. 1 presents plots of the efficient frontier estimated by SFA. For Uganda and Zambia no efficient frontier could be calculated. For these countries the regression residuals (*υ* − *μ* in equation 1) were right-skewed, and could not be decomposed into error and efficiency terms. Across countries the SFA efficient frontier is either straight or slightly convex, and qualitatively different to the concave DEA frontier.

### Efficiency scores estimated by DEA and SFA

**Fig. 2:**
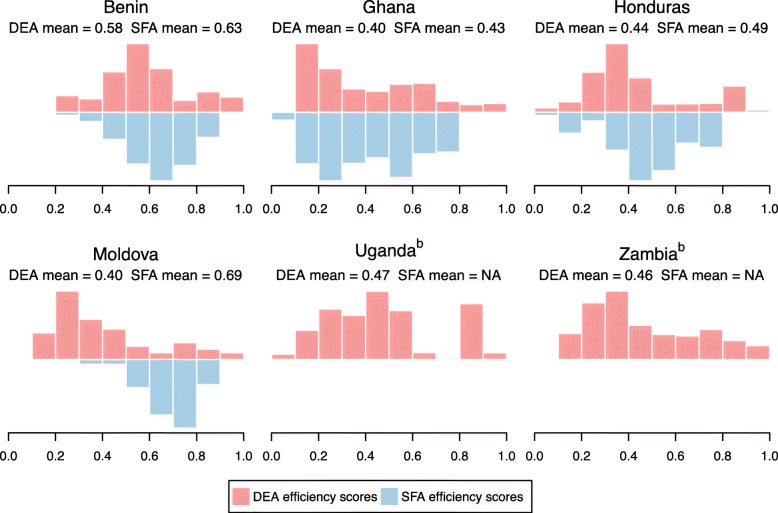
Distribution of technical efficiency scores estimated by DEA and SFA. Efficiency scores represent Shepherd input efficiency. DEA efficiency estimated by Simar and Wilson bias-corrected method under variable returns-to-scale. SFA efficiency estimated using semi-parametric SFA estimator proposed by Fan et al. Distributions and means adjusted for survey weighting. For Uganda and Zambia no frontier could be calculated, due to inability to decompose error and efficiency terms

Figure 2 shows side-by-side histograms of the distribution of DEA and SFA efficiency values (fraction of efficiency scores falling into each of 10 equally sized bins from 0.0–1.0) for each country. Average efficiency scores varied between countries, with average DEA efficiency scores varying from 0.40 in Ghana and Moldova to 0.58 in Benin, and average SFA efficiency scores varying from 0.43 in Ghana to 0.69 in Moldova. In the four countries where both scores could be calculated the mean SFA efficiency was higher than the mean value estimated by DEA, most notably in Moldova. Within each country the efficiency scores varied widely with a standard deviation of 0.18–0.23 for DEA efficiency and 0.10–0.20 for SFA efficiency.

**Fig. 3:**
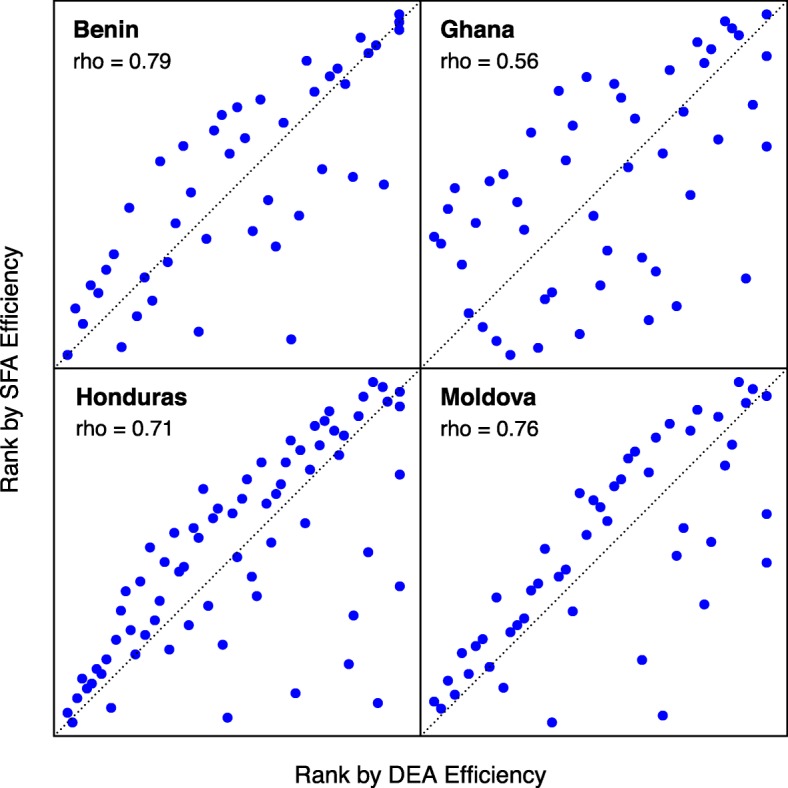
Comparison of site efficiency ranking by DEA and SFA. DEA efficiency estimated by Simar and Wilson bias-corrected method under variable returns-to-scale. SFA efficiency estimated using semi-parametric SFA estimator proposed by Fan et al. Higher rank (upper right) indicated higher efficiency score. Results for Uganda and Zambia not shown as SFA efficiency could not be estimated for these countries

Figure 3 compares the ranking of sites within each country by their DEA and SFA efficiency scores. Sites at the upper and right-hand quadrant of each panel have higher efficiency scores by SFA and DEA respectively, and sites lying on the diagonal share the same rank by both methods. We calculated Spearman’s *rho* to summarize agreement between the rankings from each score, with this rank correlation varying from 0.56 in Ghana to 0.79 in Benin. Disagreement between scores was generally higher for sites in the upper or lower tail of the distribution of service delivery costs. This is consistent with the marked difference in curvature of the efficient frontiers estimated by DEA and SFA (Fig. 1), which has a greater impact on efficiency scores for more extreme sites.

### Efficiency scores estimated by an ensemble approach

**Fig. 4:**
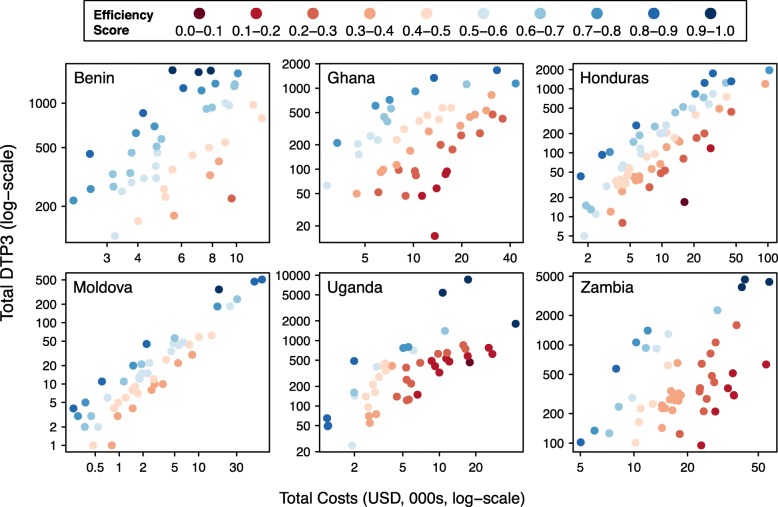
Efficiency score calculated by ensemble approach, plotted as a function of logged service delivery costs and logged service volume. For Uganda and Zambia SFA efficiency could not be calculated, and plotted values represent DEA efficiency scores

Following Di Giorgio et al. we estimated an ensemble efficiency score by averaging the DEA and SFA scores for the 4 countries with both scores available. Not surprisingly, there was relatively high agreement between this ensemble score and the DEA score, and between the ensemble score and the SFA score. Rank correlation with the ensemble score ranged from 0.88 to 0.96 for DEA efficiency and from 0.87 to 0.92 for SFA efficiency. Figure 4 plots the value of this ensemble efficiency score against logged service delivery costs and logged service volume for each country (for Uganda and Zambia the DEA efficiency is plotted).

We found a high level of agreement between the efficiency scores from the ensemble method and a crude efficiency index based on a parametric production function fitted with OLS. For the 4 countries for which ensemble results could be calculated the rank correlation with this OLS index ranged from 0.81 in Moldova to 0.92 in Benin.

### Sensitivity analysis

Table 2 presents mean efficiency scores for each method (DEA, SFA, ensemble) using different inputs (service delivery costs vs. total immunization costs) and outputs (DTP3 vs. doses delivered) per site. The rank correlation of efficiency scores with the values obtained in the main analysis is shown in parentheses. Across the six countries, scores calculated using total costs were higher than those calculated using service delivery costs (implying higher average efficiency). This likely reflects the low variability in the vaccine cost per DTP3 and per dose across sites, reducing the relative difference between most and least efficient sites. In contrast, the choice of DTP3 or doses delivered as the output measure had little impact on mean efficiency. In all countries apart from Benin there was a high level of agreement between the different approaches, with most correlation coefficients above 0.9. In Benin the results calculated using total immunization costs as the input measure differed from the main analysis, with correlation coefficients ranging from 0.62 to 0.73.

**Table 2:** Mean efficiency scores calculated with different inputs and outputs^a^

Outcome	Benin	Ghana	Honduras	Moldova	Uganda	Zambia
*Input = services delivery costs, outputs = DTP3 delivered (main analysis)*						
DEA efficiency	0.58	0.40	0.44	0.40	0.47	0.46
SFA efficiency	0.63	0.43	0.49	0.69	–	–
Ensemble efficiency	0.61	0.41	0.46	0.54	–	–
Lambda	1.44	2.18	1.54	1.02	0.00	0.00
*Input = total immunization costs, outputs = DTP3 delivered*						
DEA efficiency	0.73 (0.73)	0.48 (0.92)	0.56 (0.94)	0.45 (1.00)	0.63 (0.95)	0.55 (0.96)
SFA efficiency	0.71 (0.72)	0.43 (0.94)	0.59 (0.93)	0.68 (0.99)	0.63 (−-)	-- (−-)
Ensemble efficiency	0.72 (0.69)	0.46 (0.94)	0.58 (0.95)	0.57 (1.00)	0.63 (−-)	-- (−-)
Lambda	3.33	4.43	2.70	1.25	3.56	0.00
*Input = services delivery costs, outputs = doses delivered*						
DEA efficiency	0.60 (0.96)	0.38 (0.97)	0.44 (0.94)	0.39 (0.91)	0.47 (0.93)	0.46 (1.00)
SFA efficiency	0.58 (0.93)	0.42 (0.82)	0.61 (0.93)	-- (−-)	-- (−-)	-- (−-)
Ensemble efficiency	0.59 (0.96)	0.40 (0.93)	0.53 (0.94)	-- (−-)	-- (−-)	-- (−-)
Lambda	3.07	2.16	0.91	0.00	0.00	0.00
*Input = total immunization costs, outputs = doses delivered*						
DEA efficiency	0.74 (0.71)	0.50 (0.90)	0.53 (0.84)	0.45 (0.91)	0.58 (0.91)	0.55 (0.98)
SFA efficiency	0.76 (0.62)	0.41 (0.77)	0.64 (0.82)	-- (−-)	0.66 (−-)	-- (−-)
Ensemble efficiency	0.75 (0.65)	0.46 (0.89)	0.59 (0.85)	-- (−-)	0.62 (−-)	-- (−-)
Lambda	3.46	7.1	2.17	0.00	2.18	0.00

^a^Results for each approach (DEA, SFA, ensemble) represent mean values for each country, adjusted for survey weighting. Values in parentheses represent rank correlation of efficiency scores with values obtained for the same estimator in the main analysis. Lambda represents the diagnostic proposed by Badunenko et al.

Table 2 also reports a diagnostic described by Badunenko et al. to gauge the reliability of SFA and DEA estimates. This diagnostic (lambda) represents the ratio of the variance of *μ*_*i*_ (efficiency estimate) to the variance of *υ*_*i*_ (measurement error) calculated as part of the SFA frontier estimation. High values of lambda imply low measurement error relative to the technical efficiency, and in this context all estimators have been shown to work well [43]. When the ratio is near 1.0 results should be interpreted cautiously, and both DEA and SFA methods may underestimate efficiency. For low values of lambda most variation in the sample is due to measurement error and the efficiency results will be meaningless. For most countries and input/output combinations lambda lies in the range 1–3, implying modest confidence in the efficiency results. However, in a number of circumstances (Uganda and Zambia in the main analysis) lambda could not be estimated as the efficiency and error terms could not be statistically separated. In these circumstances, despite the fact that DEA efficiency scores could still be estimated, the results from this diagnostic suggest that little weight should be placed on the results for Zambia and Uganda.

## Discussion

We analyzed data on resource utilization and performance for 316 health facilities providing routine infant immunization services in Benin, Ghana, Honduras, Moldova, Uganda and Zambia, and used standard techniques to estimate the technical efficiency of each site compared to an efficient frontier defined by their best performing peers. Mean efficiency estimates varied by country and by analytic approach, but all lay within the range 0.4–0.7. For a given site, these input efficiency estimates represent the ratio between resource utilization under perfect efficiency as compared to observed efficiency. Subtracting the efficiency estimate from 1.0 provides an estimate of the fraction of site-level inputs that could be recovered if a site were to become perfectly efficient. Based on this definition, our study results imply a 30% to 60% reduction in costs would be possible if all sites could be relocated to the efficient frontier. This finding of low apparent efficiency levels is consistent with other studies employing similar methods to assess immunization services in low income, high burden settings [20, 21], but lower than estimates derived from a high income setting (Australia [19]). For all estimation methods there was substantial variation in efficiency scores within each country. For most countries the estimates calculated in the main analysis were robust to changes in the specification of inputs and outputs (among the options compared), although efficiency levels appeared higher when total site-level costs were used in place of service delivery costs as an input measure. The site efficiency rankings differed substantially between DEA and SFA, due to marked differences the shape of the efficient frontiers estimated by these two techniques.

Taken at face value these results imply wide variation in efficiency between sites in each country, and major opportunities to improve the overall efficiency of immunization programs through targeted intervention to resolve inefficiency in low-performing sites. However, this uncritical interpretation should be avoided. All efficiency metrics included in this analysis assume that the service delivery units being compared operate in approximately similar environments. This assumption is unlikely to hold for immunization sites, for which the catchment population may differ in size, geographic dispersion, and demand for immunization services, and which are supported by health systems of varying performance. These differences in operating environment will create variation in the resource levels required by different sites to adequately serve their target populations. SFA explicitly allows for variation in site-level performance that is not due to technical efficiency, and modern DEA approaches have similar features [36], yet both methods rely on strong assumptions in order to decompose observed variation between technical efficiency and other causes. Inability to disentangle technical efficiency and other sources of variation can bias efficiency scores downward, as has been demonstrated in simulation studies [35]. It is possible that the low apparent efficiency levels observed in this and other studies conducted in low income settings [20, 21] simply reflects variation in the operating environment of sites, and where this variation is smaller [19] apparent efficiency will be higher. In an empirical study like this it is impossible to judge the validity of the efficiency estimates against a reference standard, yet the lambda diagnostic described by Badunenko et al. provides some guidance. For Benin, Ghana, Honduras, and Moldova, this diagnostic suggests results should be interpreted with caution, while for Uganda and Zambia this diagnostic suggests efficiency results should not be trusted. While simulation studies have found the ensemble model to provide more accurate estimates of efficiency than DEA or SFA alone [35], these results will still be affected by the deficiencies of the underlying measures, and so should also be used with caution. Another reason to doubt the opportunities for substantial efficiency gains is that intervening in inefficient sites may itself be costly, though such intervention may be justified to promote equity goals.

There are several aspects of immunization program performance that were not considered in this study. The outcome used (total children receiving DTP3) is used as a proxy for completion of the infant immunization schedule, yet this is only a partial measure of the value generated by immunization programs. Achieving high coverage of the target population is another important programmatic goal, yet coverage measures available for this analysis exhibited substantial measurement error. As a consequence, even if the efficiency measures are reliable, they only provide partial evidence on the efficiency of sites in generating valued outcomes, which incorporates a broader set of concerns than simply maximizing the DTP3 per dollar. More concretely, if efforts to raise coverage increase the marginal cost of services, metrics based on the cost per output may be a poor gauge of how efficiently sites are pursing programmatic objectives. Structuring program incentives around such a metric might produce undesirable outcomes, by diverting focus away from broad program improvement towards improving a relatively narrow measure of efficiency. Moreover, as the methods examined in this study focus on inter-site variation, they will largely miss systematic factors that affect all sites simultaneously, identifying local rather than program-level inefficiencies even though the latter may be more consequential.

Despite these concerns, it is possible that some form of efficiency evaluation may play a role in program monitoring as a triage test, with sites found to have low efficiency scores marked for more intense scrutiny based on a wider set of information than the inputs and outputs considered by efficiency analysis. Conversely, sites with high efficiency scores might be studied to see whether they had successful management practices that could be adopted by other sites. For efficiency evaluation to have a role in routine program management it is inadequate to have information for a small sample of sites, and methods would need to be designed to work with the limited data routinely collected for all sites through existing reporting systems. The technical sophistication required to apply modern DEA and SFA methods might be a barrier to their use in this application, yet simpler methods might well suffice. In the 4 countries for which we were able to calculate the ensemble estimator we found a high level of agreement with a metric based on the residuals of a simple production function estimated with OLS, and this kind of simple metric might be sufficient to identify sites for further investigation. Identifying routinely reported data that adequately describe site inputs and outputs—sufficient to implement a basic efficiency analysis—could be more challenging, and this is a subject that warrants further investigation.

## Conclusions

This study applied standard efficiency estimation techniques to a large, multi-country dataset of routine infant immunization sites. Site-level results differed between these approaches, but all suggested substantial inefficiency within the sample of sites in each country. However, practical opportunities for efficiency gains are likely to be smaller than suggested by these results, with some of the apparent inefficiency reflecting measurement error, or unmodifiable differences in the operating environment, such as site location or health system structure. Adapted to work with routine reporting data and simplified methods, efficiency analysis could be used as an initial triage step to identify concerning differences between sites, which could be investigated though more in-depth investigation.

## Data Availability

The datasets supporting the conclusions of this article are available in the Dataverse repository, https://dataverse.harvard.edu/dataverse/EPIC2.

## Abbreviations

COLS: Corrected ordinary least squares
DEA: Data envelopment analysis
DMU: Decision-making unit
DTP3: 3rd Immunization for diphtheria, tetanus, and pertussis
EPIC: Expanded program on immunization costing and financing of routine immunization project
NGO: Non-Governmental Organization
OLS: Ordinary least squares
SFA: Stochastic frontier analysis
VRS: Variable returns-to-scale
WHO: World Health Organization
